# Tidying Up International Nucleotide Sequence Databases: Ecological, Geographical and Sequence Quality Annotation of ITS Sequences of Mycorrhizal Fungi

**DOI:** 10.1371/journal.pone.0024940

**Published:** 2011-09-15

**Authors:** Leho Tedersoo, Kessy Abarenkov, R. Henrik Nilsson, Arthur Schüssler, Gwen-Aëlle Grelet, Petr Kohout, Jane Oja, Gregory M. Bonito, Vilmar Veldre, Teele Jairus, Martin Ryberg, Karl-Henrik Larsson, Urmas Kõljalg

**Affiliations:** 1 Institute of Ecology and Earth Sciences, University of Tartu, Tartu, Estonia; 2 Natural History Museum, University of Tartu, Tartu, Estonia; 3 Department of Plant and Environmental Sciences, University of Gothenburg, Göteborg, Sweden; 4 Genetics, Department of Biology I, Ludwig-Maximilians University Munich, München, Germany; 5 Institute of Biological and Environmental Sciences, University of Aberdeen, Aberdeen, United Kingdom; 6 Landcare Research – Manaaki Whenua, Lincoln, New Zealand; 7 Institute of Botany, Academy of Sciences of the Czech Republic, Průhonice, Czech Republic; 8 Faculty of Science, Charles University in Prague, Prague, Czech Republic; 9 Department of Biology, Duke University, Durham, North Carolina, United States of America; 10 Department of Ecology and Evolutionary Biology, University of Tennessee, Knoxville, Tennessee, United States of America; 11 Natural History Museum, University of Oslo, Oslo, Norway; University of California Riverside, United States of America

## Abstract

Sequence analysis of the ribosomal RNA operon, particularly the internal transcribed spacer (ITS) region, provides a powerful tool for identification of mycorrhizal fungi. The sequence data deposited in the International Nucleotide Sequence Databases (INSD) are, however, unfiltered for quality and are often poorly annotated with metadata. To detect chimeric and low-quality sequences and assign the ectomycorrhizal fungi to phylogenetic lineages, fungal ITS sequences were downloaded from INSD, aligned within family-level groups, and examined through phylogenetic analyses and BLAST searches. By combining the fungal sequence database UNITE and the annotation and search tool PlutoF, we also added metadata from the literature to these accessions. Altogether 35,632 sequences belonged to mycorrhizal fungi or originated from ericoid and orchid mycorrhizal roots. Of these sequences, 677 were considered chimeric and 2,174 of low read quality. Information detailing country of collection, geographical coordinates, interacting taxon and isolation source were supplemented to cover 78.0%, 33.0%, 41.7% and 96.4% of the sequences, respectively. These annotated sequences are publicly available via UNITE (http://unite.ut.ee/) for downstream biogeographic, ecological and taxonomic analyses. In European Nucleotide Archive (ENA; http://www.ebi.ac.uk/ena/), the annotated sequences have a special link-out to UNITE. We intend to expand the data annotation to additional genes and all taxonomic groups and functional guilds of fungi.

## Introduction

Root symbiosis with mycorrhizal fungi provides fundamental benefits to plants via improved mineral nutrition and protection against diverse environmental stresses. Based on evolutionary and morphological differences, mycorrhizas are separated into four basic types, *viz.* ectomycorrhiza (EcM), ericoid mycorrhiza (ErM), arbuscular mycorrhiza (AM) and orchid mycorrhiza (OM) [1]. While the diversity and geographical distribution of host plants is relatively well-known [2], the ecology and biogeography of symbiotic fungi remains poorly understood due to their cryptic nature and high costs of identification [3].

For all types of mycorrhiza, accurate fungal identification relies on DNA sequence analysis. The nuclear ribosomal DNA (rDNA) internal transcribed spacer (ITS) region has been extensively used for species-level identification in most studies of mycorrhizal and soil-inhabiting fungi [4], [5]. ITS sequences deposited in the International Nucleotide Sequence Databases (INSD) have provided an invaluable source for inclusive, global-scale studies in *Inocybe* [6] and *Tuber* [7]. The ITS region is by far the most commonly sequenced genetic marker for molecular identification of fungi [8] except in the AM-forming Glomeromycota. In this group, the nuclear small subunit (SSU/18S) [9] and large subunit (LSU/28S) [10], [11] rRNA gene sequences are the most widely used due to exceptionally high heterogeneity of ITS copies within individual multigenomic spores.

A large proportion of the entries in INSD is not fully identified to species level (Latin binomial) or misidentified [12]. Moreover, a vast majority of the fungal entries in INSD lacks important metadata on, *e.g.*, country and region of collection, interacting taxon, and source of identification [13], [14]. In addition, many sequences are chimeric [15] or of conspicuously low read quality. Unfortunately, third-party annotation tools are poorly developed in INSD [16], which has far-reaching negative consequences for the prospects for data evaluation and filtering in large-scale sequence analyses. The mycological community has made great strides towards a better understanding of fungal taxonomy and ecology over the last few years, such that there is now a wealth of additional data and information that could be added to existing INSD sequences to cast additional light on these entries. To accomplish this in the absence of a route for direct annotation in INSD, we integrated the extended UNITE database that covers quality-checked sequences of all fungi [4] with the online annotation and search tool PlutoF [17].

Here we report on quality and metadata annotation of fungal ITS sequences deposited in INSD and downloaded to UNITE. The associated quality tags and metadata were introduced to specific data fields of UNITE and are publicly available for search and rapid download via the UNITE homepage (http://unite.ut.ee/). We aim to extend the annotation of fungal INSD sequences by including experts on non-mycorrhizal taxa and cover additional genes. Our integrated annotation and query platform facilitates data mining of quality-checked taxonomic, ecological and biogeographic information, and provides important insights into the biodiversity and ecology of mycorrhizal as well as other fungi.

## Materials and Methods

**Figure 1:**
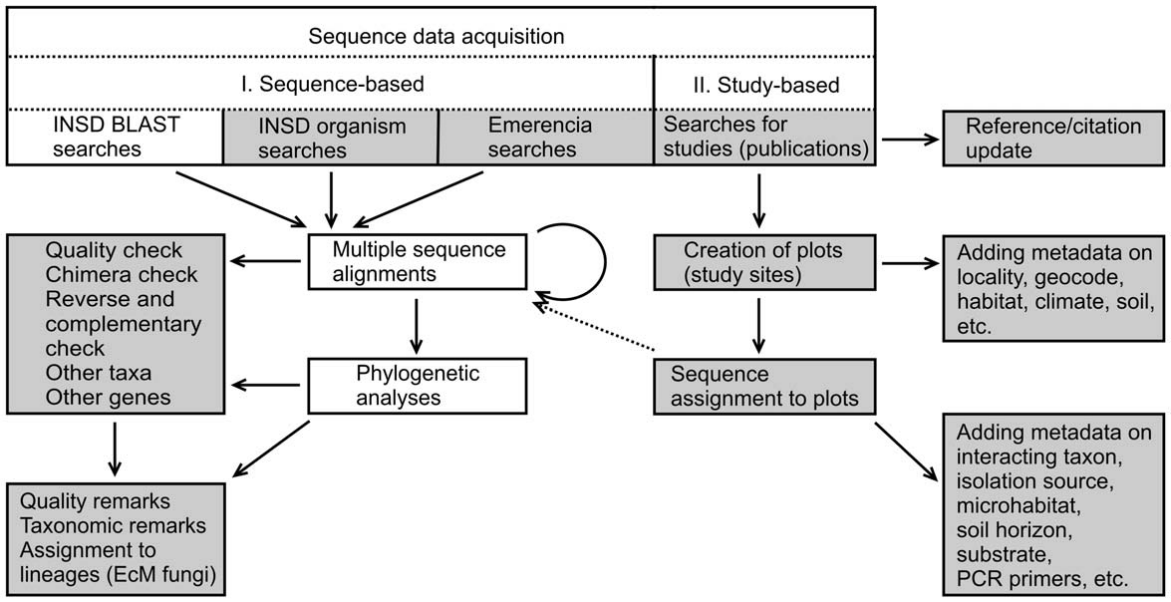
Scheme of the metadata annotation workflow. Shaded boxes indicate procedures performed and/or saved over the PlutoF workbench (http://plutof.ut.ee/).

All new fungal ITS sequences (annotated as such in INSD) are downloaded from INSD to UNITE on a bimonthly basis. The present work reports on the annotation of ITS sequences that were publicly available as of January 18, 2011. Very short sequences (<200 bp.) and sequences derived from Next Generation Sequencing techniques – that are normally not allowed in INSD – were excluded. Sequences were annotated by experts on particular mycorrhizal types and/or taxonomic groups. Annotations of quality and metadata were performed in two steps – on the basis of taxonomy and the corresponding scientific study (Figure 1).

### Sequence annotation by taxonomic groups

For EcM, AM, ErM and OM fungi, all representatives of the major mycorrhizal taxonomic groups were retrieved through the use of names of the inclusive taxa as search strings in the organism field in the PlutoF workbench. Unnamed sequences (i.e. ‘uncultured fungus’) were retrieved by running BLASTn searches and emerencia searches (i.e. searches for sequences that represent unidentified organisms [18]) against INSD using several randomly selected representative sequences or fully identified species as proxies [14]. These taxonomic groups represent lineages of EcM fungi [19] and cover several approx. family-level clades of Helotiales, Sebacinales, Chaetosphaeriales, Chaetothyriales and Cantharellales for ErM and OM fungi. All sequences putatively belonging to these taxa were downloaded via PlutoF workbench and were subsequently subjected to multiple alignments with MAFFT 6.6 (http://mafft.cbrc.jp/alignment/server/index.html). The annotations for AM fungi are based on a manually curated sequence alignment and database that covers the SSU, ITS and LSU sequences (A. Schüβler *et al*., unpublished). The taxonomic affiliations of Glomeromycota sequences were revised according to [20]. All alignments were inspected by eye to identify slowly and rapidly evolving regions within the ITS sequences. All sequences that were poorly aligned to other species were subjected to bulk megablast searches against INSD and UNITE as implemented in the PlutoF workbench. This enabled us to identify potentially chimeric and reverse complementary sequences as well as sequences belonging to non-targeted taxa. Most of the chimeric and low-quality sequences were discovered by carefully inspecting the alignment, followed by separate BLASTn searches of ITS1 and ITS2 against INSD to confirm their chimeric status [15]. Potentially low-quality sequences were primarily recognized as sequences with unique gaps and indels in the conserved regions, especially in the 5.8S gene, as compared to their closest sequences. Sequences were also considered of low quality when the beginning or end of the ITS spacers contained >2 obvious substitution errors or indels resulting from inadequate end trimming. Sequences containing ambiguous bases were not automatically treated as of low quality, because polymorphism in ITS alleles is not uncommon in the dikaryotic Basidiomycota [21]. These sequences were, however, carefully checked for other indicators of low quality, because ambiguous bases may equally well arise from low-resolution sequence chromatograms. Several putatively low-quality sequences originated from our own laboratories; therefore we had the opportunity to re-check the sequence chromatograms. Of these, only a few divergent tropical sequences were regarded as false positives in terms of low quality assignment, which provides reasonable support for our subjective decisions in general. Sequences passing the quality control steps were re-aligned with MAFFT; the alignments were corrected manually and subjected to Maximum Likelihood analyses using RAxML ([22]; http://phylobench.vital-it.ch/raxml-bb/) or PhyML ([23]; http://www.bioportal.uio.no/appinfo/show.php?app=phyml) with default options. Sequences with disproportionately long branches were, once again, checked for potential chimeric insertions and low quality.

### Sequence annotation by studies

Both in INSD and PlutoF, the concept of a ‘scientific study’ constitutes a fundamental data unit. Studies comprise all sequences that were submitted by the same author(s) under the same study title, and they usually represent a single published or unpublished article. Because approx. 10–20% of the unnamed sequences were not retrieved in the taxonomy-based approach, we downloaded all sequences by studies (excluding those with <10 sequences). In this step, metadata on isolation source, locality and interacting taxon (host) were annotated. Whenever necessary, the original publications were examined and the corresponding authors were contacted for additional information.

All EcM-derived sequences that lacked lineage annotation were subjected to further bulk megablast searches. If necessary, multiple alignments were constructed to evaluate the decisions. This enabled us to assign further sequences to EcM lineages or groups of non-ectomycorrhizal or uncertain trophic status. For ErM and OM fungi, a note was added whether or not this fungus forms mycorrhizal structures such as coils or pelotons, and/or stimulates plant growth in pure culture synthesis trials.

## Results and Discussion

### Sequence reliability

As of January 18, 2011, INSD comprised 183,208 fungal ITS sequences. Of these, 28,791 (15.7%) sequences belonged to EcM fungi and 3,176 (1.7%) to AM fungi (Table 1). In total, 1,457 (0.8%) and 2,267 (1.2%) sequences were recovered from roots of ErM plants and orchids, respectively.

**Table 1:** Quality and metadata annotations of fungal ITS sequences by mycorrhiza types.

		EcM fungi^1^	AM fungi^2^	ErM fungi^3^	OM fungi^3^	All fungi
Studies	INSD original	1242	127	78	93	12004
Fungal ITS sequences	INSD original	28791	3176	1457	2267	183208
Chimeric sequences^4^	INSD original	nd^5^	nd	nd	nd	nd
	Annotated^6^	nd	nd	46	11	677
Low-quality sequences	INSD original	nd	nd	nd	nd	nd
	Annotated	1970	25	56	121	2572
Sequences annotated for country	INSD original	16390	2319	751	1150	104426
	Annotated	21891	2603	1253	2053	111647
Sequences annotated for geocode	INSD original	2276	271	163	91	11331
	Annotated	9566	990	518	696	30702
Sequences annotated for interacting taxon (host)	INSD original	6272	835	1093	1608	43126
	Annotated	8890	2337	1455	2165	52551
Sequences annotated for isolation source	INSD original	10891	2274	991	1335	30030
	Annotated	27686	3000	1456	2209	79353
Sequences/isolates with experimental evidence for function^7^	INSD original	nd	nd	nd	nd	nd
	Annotated	nd	nd	226	140	nd

1 all sequences belonging to EcM lineages regardless of isolation source;

2 all sequences belonging to Glomeromycota, except *Geosiphon;*

3 all sequences derived from roots (with or without a culturing step) of the respective host plants;

4 Chimeric sequences consist of two or more fragments of fungal sequences and are therefore not assigned below the kingdom level;

5 nd, not determined;

6 Annotated–sum of original annotations and metadata provided in the course of this study;

7 formation of coils in ErM, formation of pelotons, stimulation of germination or development in OM.

Sequence quality was evaluated based on 170 alignments that covered virtually all EcM, AM, ErM and OM taxa. Based on these alignments, we identified and annotated 2,172 (6.1%) mycorrhizal sequences of potentially low quality. These low-quality sequences were particularly abundant in studies published in 1990s and in studies that used one of the ITS1, ITS3 or ITS4 primers as the only sequencing primer. These primers are located close to or within (ITS3 primer) the ITS region [24] and the first tens of basecalls are often unreliable. More conservative trimming of the low-quality ends would clearly ameliorate this problem. Cloning methods often reveal abundant single nucleotide polymorphisms and indels [25], which we observed uniformly distributed across the ITS region, including the relatively conserved 5.8S gene and motifs within the spacer regions. Therefore, we argue that many cloning-based estimates of intragenomic sequence variation [21], [26] are in fact over-estimates due to the potential PCR or cloning errors. Different rDNA copies may, however, occur within multigenomic spores of Glomeromycota [11] and certain higher fungi [27], where some alleles may have lost function due to redundancy.

Sequences of clones derived from diverse habitats such as roots and particularly soil constitute a major source for chimeras. Chimeric entries are a major problem in some studies, the most extreme one featuring 123 chimeras. Eleven studies comprised >10 chimeric sequences that accounted for 8.5% entries on average (range, 3.1–24.6%). With a few exceptions, chimeric sequences were singletons (but see ref. [28] for the evolution and distribution of chimeric sequences in SSU of prokaryotes). Information from alignments, phylograms and separate blast searches for ITS1 and ITS2 against INSD revealed further chimeras and confirmed those recognized by the program Chimerachecker [15]. Chimeras of two species of Helotiales were relatively common among the soil clone sequence entries. We also detected chimeras that were formed between two sister species of *Wilcoxina* and two species of *Piloderma*. Because singletons and doubletons form a basis for the non-parametric minimum species richness estimators, chimeric and low-quality sequences may strongly bias these estimates upwards [29]. In addition to clone libraries, chimeras were sometimes formed during erroneous contig assembly, where the constituent fruit body sequences clearly originated from different taxa. This study does not cover chimera formation between SSU and ITS or ITS and LSU regions. The proximate ends of these flanking rRNA genes are probably critical sites for chimera formation, because of their highly conserved structure. The proportion of chimeric sequences probably exceeds 1.5% (as estimated in [15]), because data from error-prone massive cloning studies and Next Generation Sequencing studies are rapidly accumulating. In addition, chimera formation is more likely between closely related taxa [28] that render their automatic detection difficult [15].

### Metadata and experimental functions

Consistent with Ryberg et al. [14], we found fungal sequences from all mycorrhizal types to be poorly annotated with metadata such as interacting taxon (host), locality and isolation source. Submission of a single representative sequence for the entire study emerged as a major problem, because information on its precise source was lacking or ambiguous due to its composite nature. To retain the information of multiple host and soil associations, we created extra data fields–‘additional host’ and ‘additional soil’–where the alternative options were inserted as text entries. Alternatively, sequence entries can be duplicated in PlutoF–i.e. dummy accessions are created to account for all possible combinations in the metadata of sequence entries [9].

Information on country and geocode (i.e. latitude and longitude) were available for 20,610 (57.8%) and 2,801 (7.9%) sequences across all mycorrhiza types, respectively. Data on host and locality were particularly scarce for sequences originating from fruit bodies. Old collections of fruit body specimens are often equipped with ‘soil’ or ‘mosses’ as substrate and ‘forest’ or ‘road side’ as biotope. Mycologists have traditionally relied on morphology of fruit bodies and spores rather than potential hosts when separating species. The lack of locality data in fruit body-derived entries may be rooted in the fact that specimens used for taxonomic studies are collected from various locations, and it is time-consuming to check and enter collection details one by one in the data submission window of INSD. However, accurate information about the locality of specimens is often lacking in the original publication as well. By examining various sources, we retrieved the missing information on country and geocode for 7,190 (20.1%) and 8,969 (25.1%) sequence entries of mycorrhizal fungi, respectively.

Metadata on interacting taxon were available for 6,272 (21.8%), 835 (26.3%), 1,093 (75.0%) and 1,608 (70.9%) entries of EcM, AM, ErM and OM fungi, respectively (Table 1). We considered a correctly spelled Latin binomial or genus name sufficient for annotation. Scant information is partly attributable to uncertainty, because natural plant communities are usually diverse, and because spatial and temporal variations in the life cycle of fungi can hinder our ability to link them to a particular host plant. For example, hyphae and fruit bodies of EcM fungi may extend far away from the host plant [30]. Similarly, basidiospores of some EcM fungi (e.g. *Rhizopogon*) readily germinate and colonize seedlings after being dormant for several years [31]. Spores of Glomeromycota may remain dormant in agricultural soils long after the original crop has been replaced. In AM-fungi, we separated the natural hosts from laboratory bait hosts, which are selected due to their ease of manipulation. The laboratory bait hosts are given in a separate remarks field. We updated or added information about interacting taxon (host) to 5,039 (14.1%) entries of mycorrhizal fungi taken together.

The availability of information on isolation source varied greatly among mycorrhizal types (Table 1). Submitters of fungal ITS sequences used 5,349 different terms or phrases to characterize the isolation source. We reduced this multitude of variants into 20 options (air, animal sample, DNA from wood, dust fungal DNA, ectomycorrhiza, ericoid mycorrhiza, fruitbody, fungal mycelium (ingrowth bag), leaf litter, lichen, orchid mycorrhiza, plant bark, plant fruit, plant leaf, plant root, plant seed, soil fungal DNA, soil fungal RNA, spore, water) that are applicable to nearly all fungal samples. Further specifications can be provided in the remarks field. We considered the information about isolation source unambiguous and informative for 15,491 (43.4%) sequence entries of mycorrhizal fungi. We updated or added source information to 18,860 (52.8%) entries. In AM fungi, plant roots, spores and soil contributed 41.2%, 33.9% and 19.0% to the source of isolation, respectively. In EcM fungi, fruit bodies, ectomycorrhizas and soil DNA accounted for 43.3%, 32.4% and 14.6% of the identification sources, respectively. There were substantial differences in the proportions of these sources among EcM fungal lineages, reflecting the disparity in insights into EcM fungal biodiversity as based on fruit bodies, root tips and mycelium (Table 2). Some lineages with predominately contact exploration type of EcM and sparse emanating mycelium-such as the /russula-lactarius, /clavulina and /hygrophorus [32]-were relatively common among the soil-derived sequence entries. Whether these conflicting patterns are truly biological or attributable to PCR bias remains unsettled.

**Table 2:** Contribution of different isolation sources to the number of INSD sequences in the most common ectomycorrhizal fungal lineages.

Lineage	Fruit-body	Ectomycorrhiza	Soil and mycelium	Total
/russula-lactarius	1772	1638	1238	5023
/cortinarius	2714	1001	363	4241
/tomentella-thelephora	310	1454	478	2840
/inocybe	1126	470	399	2139
/tuber-helvella	1410	285	37	1828
/suillus-rhizopogon	744	610	119	1552
/boletus	697	213	33	984
/hebeloma-alnicola	422	174	97	864
/tricholoma	466	172	125	862
/amanita	532	118	41	732
/pisolithus-scleroderma	458	77	43	702
/piloderma	14	390	227	657
/sebacina	29	358	50	592
/cenococcum	0	408	45	515
/amphinema-tylospora	2	284	163	468
/clavulina	58	159	108	414
/wilcoxina	2	119	249	404
/cantharellus	123	193	25	361
/laccaria	83	158	71	352
/ramaria-gautieria	214	33	7	275
/hydnellum-sarcodon	219	13	5	238
/terfezia-peziza depressa	104	69	29	221
/meliniomyces	0	144	31	218
/paxillus-gyrodon	72	45	5	206
/genea-humaria	74	101	5	199
/geopora	124	35	7	180
/albatrellus	132	20	3	156
/hygrophorus	67	27	48	146
/pseudotomentella	16	88	12	129
/phellodon-bankera	119	2	6	128
/tomentellopsis	31	57	12	109
All lineages	12465	9315	4214	28791

In contrast to EcM and AM mycobionts, the fungi inhabiting roots of ericoid plants and orchids were identified directly from roots with or without a culturing step. In putatively ErM fungi, 690 (47.4%) sequences were obtained directly from ErM roots and 767 (52.6%) sequences were obtained from living cultures. In the cultured isolates, we could trace the symbiotic performance of 226 isolates in various experiments. Taken together, 60.2% of the isolates were capable of forming coils and/or stimulating growth of ericoid plants *in vitro.* More than 95% of the functional ErM mycobionts belonged to the Helotiales. Cultures identified as Hypocreales and Coniochaetales probably represent fast-growing contaminants, because these taxa have never been suggested as functional partners in ErM. In OM fungi, 1,591 (70.2%) and 676 (29.8%) sequences originated from orchid mycorrhizas and living cultures, respectively. Out of the 168 experimentally tested isolates, 139 (82.7%) stimulated seed germination or growth of host plants.

While taxa from all fungal phyla have been identified from roots of orchids and ericoid plants, experimental evidence for functional association covers only a few, albeit large, groups of fungi. The remaining DNA isolates may belong either to unculturable mycorrhizal fungi or to non-mycorrhizal guilds of opportunistic pathogens, endophytes or saprobes [33], [34]. As an alternative to direct synthesis experiments, electron microscopy may provide in situ evidence for functional associations between plants and fungi at higher taxonomic levels [35]. For example, electron microscopy has confirmed that certain members of Atractiellomycetes form pelotons in orchid roots [36] and that Serendipitaceae form coils in ErM roots [37]. Many orchids have switched fungal partners from the common soil saprobes (the *Rhizoctonia* form genus) to EcM fungi that are particularly difficult to obtain into pure culture. Therefore, experiments with these fungi are disproportionately rare. Overall, both ErM and OM formation appeared inconsistent among strains of individual phylogenetic species [33], [38], but this may possibly reflect variation in experimental conditions, including conditions that are non-optimal for mycorrhiza formation.

### Benefits and implications

**Figure 2:**
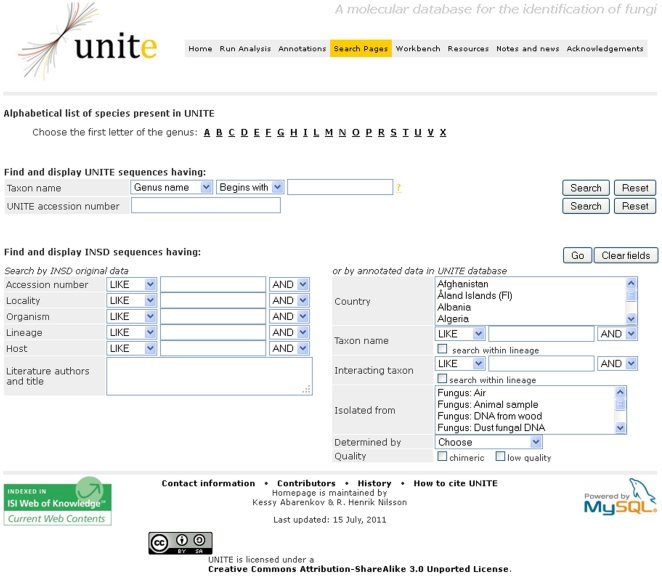
Screenshot of the UNITE search page (http://unite.ut.ee/). This search tool allows queries of sequences based on taxonomic information and associated metadata.

This study reports on the annotation of sequence quality and addition of metadata to the existing INSD entries. These are publicly available for search, download and use for subsequent analyses via the UNITE database (Figure 2). The steps of quality filtering and supplementation of metadata to sequence entries form an integral part for any large-scale phylogenetic and biogeographic analyses [6], [39]. In large datasets, erroneous single nucleotide indels may severely distort multiple alignments, because these are constructed from blocks of subalignments comprising the closest related sequences. Currently available alignment programs seem to have difficulties aligning these gap-rich blocks against each other [40].

The approach presented here for annotating existing sequence data differs fundamentally from that of quality-filtered, narrow-niche fungal sequence databases represented by the first version of UNITE, PHYMYCO-DB [41], Fusarium-ID [42] and the prokaryote databases GreenGenes [43] and SILVA [44]. These databases comprise the narrow selection of sequences regarded as high quality and excluding vast majority of others. Incorporating INSD sequences to these databases is relatively slow and relies on manual action by members of one or a few research groups. We advocate that the strengths of our approach lie in the i) automation of sequence data download from INSD and ii) contribution of annotations from many institutionally unrelated expert fungal taxonomists and molecular ecologists over our mirrored web server [17]. Users are required to register for a username and password, because annotations are non-anonymous in the interest of reliability.

In addition to these benefits, there are various risks associated with management of such platform. First, there will always remain a backlog of unchecked, newly published sequences that can be naively used as good-quality data. Second, the database may deteriorate if researchers take little interest in making their expert work publicly available. Therefore, the workbench is intended for multifunctional development according to the needs of the users, including submission and sharing of unpublished data among workgroups and running analyses [17]. In addition to various search options by taxonomy and study, the updated version of UNITE allows users to search for country, interacting taxa, remarks on ErM and OM function, etc. The results are returned in a spreadsheet format and can be easily sorted by any criterion.

In conclusion, the annotated metadata for locality and interacting taxa facilitate the undertaking of large-scale studies in mycorrhizal data mining, fungal biogeography and phylogenetic community composition. Flagging low quality and chimeric data improves the reliability of fungal diversity estimates based on molecular data and enables construction of automated species identifiers. Since August 2011, the added metadata has been made available via a link-out function in the European Nucleotide Archive (ENA; http://www.ebi.ac.uk/ena/), which further eases access to the annotated data. Further annotation of sequence data will depend on additional expert users to address taxonomy, biogeography and biodiversity of fungi as part of their every-day research. Thus, we welcome any such contribution by the readers of the present study. With minor modifications, this platform could be extended for annotating any gene in any group of organisms.
